# Case report: A rare case of recurrent right atrial mass dramatically disappeared after anticoagulation

**DOI:** 10.3389/fcvm.2022.1066065

**Published:** 2022-11-10

**Authors:** Huarui Wang, Jie Zi, Qiao Li, Yuanyuan Sun, Ling Zhu

**Affiliations:** ^1^Department of Respiratory and Critical Care Medicine, Shandong Provincial Hospital Affiliated to Shandong First Medical University, Jinan, China; ^2^Department of Cardiac Surgery, Shandong Provincial Hospital Affiliated to Shandong First Medical University, Jinan, China; ^3^Department of Cardiac Ultrasound, Shandong Provincial Hospital Affiliated to Shandong First Medical University, Jinan, China

**Keywords:** right atrial mass, pulmonary embolism, anticoagulation, thrombophilia, thrombomodulin

## Abstract

A young man repeatedly found a right atrial mass with severe wheezing and extreme dyspnea. His condition was critical and complicated. The process of correct diagnosis was full of twists and turns. Finally, he got better and was discharged from the hospital after anticoagulation therapy, which suggested that correct clinical thinking and decision are particularly important in the process of diagnosis and treatment.

## Introduction

The detection rate of right heart thrombosis in patients with acute pulmonary embolism is 4–18%, which can occur in the right atrium, tricuspid valve, right ventricle, patent foramen ovale, and other sites (1). Echocardiography is the main diagnostic method for right heart thrombosis, but it still needs to be differentiated from other diseases such as myxoma. Although pulmonary embolism combined with right heart thrombosis is rare, its mortality is high and cannot be ignored clinically.

## Case presentation

**FIGURE 1:**
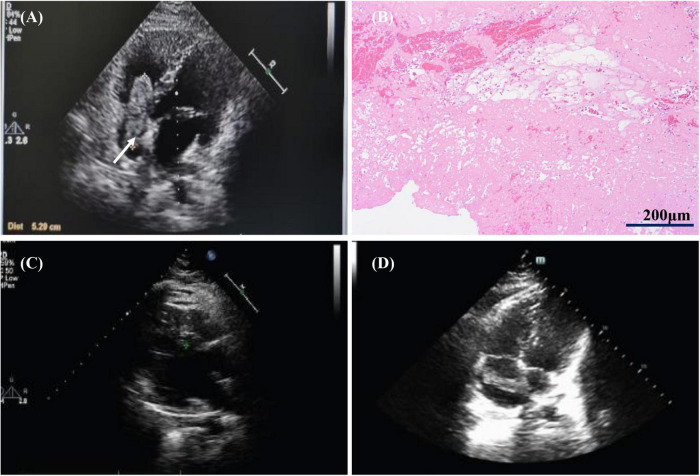
Images of first hospitalization and recurrence of right atrial mass. **(A)** The first echocardiography revealed a right atrial mass. **(B)** Pathological image of right atrial mass: a small number of platelets and inflammatory exudates. **(C)** Echocardiography at 10 days after operation showed that the right atrial mass disappeared. **(D)** Right atrial mass revisited (Less than one month after surgery) White arrows represent thrombus.

The patient was a 26-year-old male who presented to the Department of Cardiac Surgery with chest tightness and dyspnea. He had had a poor spirit, dyspneic appearance, heart rate 115/min, respiratory rate 30/min, blood pressure 106/92 mmHg and oxygen saturation 90%. Physical examination showed cyanosis, the breath sounds of both lungs were thick on auscultation, and a little phlegm rale could be heard. No obvious abnormality in other physical examinations. Laboratory test: D-dimer (0.9 mg/L), the chest CT showed some small pulmonary infarct areas in both lungs that were ignored by us until we made a correct diagnosis. The echocardiography found the right atrium mass (2021.06.21). It showed a long oval moderate echo mass in the right atrium with a length of about 5.29 cm and a less smooth surface, which seemed to be pedicled attached to the right atrioventricular orifice of the inferior vena cava (Figure 1A). The range of motion was large with the cardiac cycle, and the diastolic mass could be removed to the tricuspid orifice, and the systolic light mass returned to the right atrium. It is suggested that the solid space occupying in the right atrium, considering the right atrial myxoma, he was admitted to the Department of Cardiac Surgery. The pathological examination of the extract showed fibrinoid necrosis (Figure 1B), in which a small number of platelets and inflammatory exudates were seen, no obvious tumor cell components were found, and the total volume was 6 cm × 2 cm × 1.5 cm. Conventional anticoagulant therapy was given post-operatively. Ten days after surgery, the symptoms were significantly improved, no abnormal mass-like echo was detected in the cardiac cavity by echocardiography (Figure 1C). The patient was discharged from the hospital with warfarin (INR 2-3). However, less than a month after surgery, the patient came to the Department of Cardiac Surgery again due to dyspnea. The right atrial mass was revisited (3.41 cm × 0.81 cm, Figure 1D).

The patient’s condition is recurrent for a short period of time, which is caused by heart failure, arrhythmia, tricuspid valve injury, embolism, and other complications after cardiac surgery, or is there another reason in the initial diagnosis? We urgently improved the relevant auxiliary examination and found that the patient had elevated D-dimer (2.13 mg/L), hypersensitive troponin T 16.7 pg/ml, echocardiography showed that the right atrial mass appeared again.

**FIGURE 2:**
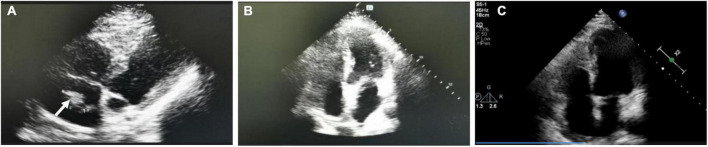
Echocardiography during the second hospitalization and follow-up period. **(A)** Right atrial mass became less after a week of anticoagulation. **(B)** Right atrial mass disappeared completely after 2 weeks of bridging anticoagulant therapy. **(C)** Echocardiography showed no right atrial thrombus (Follow-up period).

**FIGURE 3:**
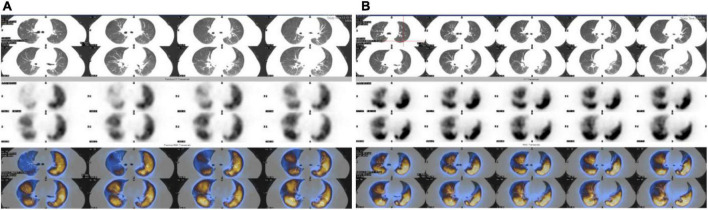
Pulmonary ventilation and perfusion imaging before and after treatment. **(A)** Pulmonary embolism was confirmed by pulmonary ventilation and perfusion imaging. **(B)** Pulmonary ventilation and perfusion imaging was normal (Follow-up period).

The patient was admitted to the Department of Cardiac Surgery for the second time, we asked the following question: Was this reappeared right atrium mass myxoma or thrombus? If there was a thrombus in the right atrial, where does the thrombus originate? In view of the patient’s repeated and critical condition, we organized multidisciplinary expert consultation. Respiratory expert pointed out that the patient had repeated dyspnea and chest tightness, echocardiography showed a mass in the right atrium one month ago, so the diagnosis of myxoma was excluded. Therefore, the right atrial mass was considered to be a thrombus rather than a myxoma. The active thrombus in the right atrium has the potential to form a fatal pulmonary embolism. It is necessary to perform computer tomography pulmonary angiography (CTPA) examination as soon as possible to confirm the diagnosis, actively screen for thrombogenic factors, and immediately perform empirical anticoagulation therapy. Experts in rheumatology, immunology and hematology pointed out that they agreed with the respiratory experts. The antinuclear antibody spectrum, antiphospholipid antibody, lupus index, humoral immunity, and lower limb vascular ultrasound were normal, and the patient was allergic to iodine contrast media in the past, so CTPA examination could not be performed. At the time, the patient had worsening dyspnea, cyanosis, unable to lie down, heart rate 124/min, respiratory rate 26/min, blood pressure 117/78 mmHg. The hemodynamics of the patient was stable and was treated with empirical anticoagulation therapy (enoxaparin 60 mg/12 h, subcutaneous injection; warfarin 2.5 mg/day, orally), and the relevant blood coagulation indexes were monitored, the hemodynamic changes were closely monitored, and the patient’s condition was evaluated all the time. Once the condition increased to high-risk, thrombectomy should be performed immediately. After a week of anticoagulation therapy, the patient’s symptoms such as dyspnea were significantly improved, and D-dimmer decreased to 1.18 mg/L, and the right atrial mass became smaller (2.66 cm × 1.56 cm) (Figure 2A) and pulmonary arterial systolic pressure from 37 mmHg decreased to 29 mmHg, anticoagulant therapy had an immediate effect, which also confirmed the possibility of right atrial thrombosis. Then we chose pulmonary ventilation and perfusion imaging, confirming pulmonary embolism and right atrial thrombosis (Figure 3A). After 2 weeks of bridging anticoagulant therapy, echocardiography showed that the right atrial mass disappeared completely (Figure 2B), and the patient was discharged from the hospital. Warfarin was used to continue the anticoagulant therapy, INR2-3 was maintained, during follow-up, echocardiography and pulmonary ventilation and perfusion imaging showed significant improvement in both right atrial thrombosis and pulmonary embolism (Figures 2C, 3B).

The patient was a young male with a healthy past and no family history of thrombosis. He had repeated right atrial thrombosis in a short period of time, but the relevant thrombogenic tests were all negative. In order to determine the cause of the disease, a thrombogenic gene test was carried out, and the results showed *thrombomodulin gene* (*THBD*) mutation (Chr20:23028799, Exon1, p. C455R). He was considered to be a patient with thrombophilia.

## Discussion

This case was a young male patient with recurrent right atrial mass complicated with pulmonary embolism. The etiology is complex and the diagnosis and treatment process is difficult. We consider the hypercoagulable state caused by *THBD* mutation that was the main cause of this patient’s recurrent thrombus in the right atrium rather than the complication of surgery. When the patient is discharged, anticoagulant therapy is continuous which prevents thrombosis as a complication after cardiac surgery. Right atrial thrombosis occurs in 4–8% of patients with acute pulmonary embolism (2). The clinical cases are rare, but the mortality rate is high. For this kind of patient, time is of the essence, and correct clinical thinking and bold decisions are particularly critical.

Right atrial thrombus is mostly metastatic thrombus from peripheral deep vein, and most patients have a long history of immobilization. The right atrium can also form thrombus at the original location due to endocardial injury, blood hypercoagulability, endothelial cell injury and organic heart disease, such as right heart failure, atrial fibrillation and right atrial ventricular valve replacement, indwelling catheter, pacemaker, etc (3, 4). In our patient, the possible cause is a hypercoagulable state caused by his genetic mutation.

Echocardiography is often used for the diagnosis of cardiac mass, but it cannot determine the nature of the mass. It is difficult to distinguish atrial myxoma from atrial thrombosis. Relevant cases have shown that both of them can occur in the left or right atrium, presenting an oval shape, and can be attached to the atrial septum, with pedicles, mobility, and calcification (5). Cardiac MRI performs better than echocardiography at determining the nature of cardiac lesions and can differentiate myxomas from thrombus (6). However, the patient’s condition was too severe to go out for examination. After anticoagulation treatment, the right atrial mass was smaller than before, which verified the diagnosis of right atrial thrombosis. A total of 5 cases of myxoma-like right atrial thrombosis in the past 10 years from 2011 to 2021 were screened out through PubMed, including 4 elderly patients and 1 young patient, showing different symptoms such as palpitations, dyspnea and syncope (7–11). All patients had thromboembolic causes of cardiac thrombosis, including cardiac structural abnormalities, catheter-related thrombosis, pacemaker implantation, and long-term bed rest history. Three of them were diagnosed with right atrial thrombosis by post-operative pathology, and the other two patients could not be surgically resected, and the right atrial mass disappeared after empirical anticoagulation. Compared with the above patients, our patient is a young man who had no easy thrombosis cause of cardiac thrombosis in the past, was misdiagnosed as myxoma for the first time and underwent surgical treatment, and had a recurrence of right atrial mass in a short time, taking into account the possibility of right atrial thrombosis. This warns clinicians that when they encounter patients with cardiac space occupying, they should be on guard against the possibility of thrombosis so as not to delay diagnosis and treatment. Our patient’s genetic testing revealed *THBD* mutation. The *THBD* encodes thrombomodulin (TM). TM deficiency or dysfunction leads to a prethrombotic state or venous thrombotic hypercoagulation state. Manderstedt et al analyzed the exome sequences of *THBD* for qualifying variants in 28,794 subjects found that rare qualifying *THBD* variants were associated with venous thromboembolism (12). *THBD* mutation in this patient would induce endothelial cell shedding, increase the level of prothrombin in the circulation, affect the level of activated protein C (APC), and then affect the coagulation regulation effect of APC proteolytically inhibits the coagulation cofactors Va and VIIIa, aided by protein S. In this patient, we found the protein C decreased. And finally, it will result in the patient’s blood hypercoagulation state. This further provides an explanation for the patient’s recurrent right atrial thrombosis.

At present, there is a lack of evidence-based medical research on the treatment of right atrial thrombosis combined with pulmonary embolism, and its treatment principle is mainly based on case reports. Multiple studies have shown that patients with moderate and low risk are more suitable for anticoagulant therapy (13). Barrios et al. (14) prospectively analyzed 325 patients with pulmonary embolism complicated with right atrial thrombosis and found that thrombolysis and surgical thrombectomy did not significantly improve the survival prognosis of patients compared with anticoagulant therapy, and had a higher risk of recurrence. In a study of 328 patients with pulmonary embolism and right heart thrombosis, among hemodynamically unstable patients, the survival rate was higher in the thrombolytic group (81.5%) than in the surgical thrombectomy group (70.5%) and anticoagulation group (47.7%) (15). Based on the previous studies, our patient was hemodynamically stable and improved significantly with anticoagulant therapy, avoiding the trauma of surgery. There are three types of right heart thrombus (Table 1) (1, 2). According to relevant guidelines and studies if thrombolytic therapy is contraindicated or ineffective in hemodynamically unstable patients, surgical thrombectomy is recommended. Besides, the larger type A thrombus; larger type C thrombus with potential risk of blocking the right atrium or right ventricular outflow tract; right heart thrombus riding in the foramen oval are recommended for surgical thrombectomy (2, 3, 13). we considered that the right atrial thrombosis of the patient from the inferior vena cava thrombus. His hemodynamics was stable, accompanied by myocardial injury, which was in line with middle-risk pulmonary embolism combined with right atrial thrombosis. According to the studies, anticoagulant therapy was given. At the same time, we closely monitor the hemodynamic changes of the patients, prepare for the deterioration of the patient’s condition, start the multi-disciplinary team at any time, and remove the thrombus if necessary. Fortunately, the anticoagulant effect of the patient is good, which provides experiences for the diagnosis and treatment of such rare cases.

**TABLE 1:** Types and characteristics of right heart thrombosis.

Types	Characteristics
Type A thrombus	Active thrombus, high early mortality, is mainly migrated from the peripheral venous system, similar to wormlike, which is closely related to clinical severe pulmonary embolism.
Type B thrombus	Static non-specific thrombus, low early mortality, which is mainly caused by right ventricular dilatation resulting in blood stasis that is caused by atrial fibrillation and tricuspid stenosis. The risk of clot shedding is relatively small.
Type C thrombus	This is an active thrombus partially attached to the lumen, intermediate in character of A and B, which has a potential risk of blocking the right atrium or right ventricular outflow tract.

## Conclusion

We report a case of recurrent right atrial thrombosis with pulmonary embolism. The patient’s condition repeated and the diagnosis process was bumpy. When patients with right atrial mass occur, clinicians should consider the possibility of right heart thrombosis. This case provides corresponding experience and lessons for clinical workers. We should actively screen the factors of thrombolysis, make early diagnosis, closely monitor the coagulation indexes and hemodynamic changes, and start anticoagulation as early as possible. Importantly, we should activate the strength of multidisciplinary team in some complicated cases.

## Data availability statement

The original contributions presented in this study are included in the article/supplementary material, further inquiries can be directed to the corresponding authors.

## Ethics statement

Written informed consent was obtained from the individual(s) for the publication of any potentially identifiable images or data included in this article.

## Author contributions

HW, YS, and LZ conceived the presented idea. HW wrote the manuscript. JZ did the operation. QL provided the photos. LZ and YS revised the manuscript. All authors have read and approved the final version of the manuscript.
